# Effectiveness of Manual Bronchial Clearance Techniques in the Treatment of Bronchiolitis

**DOI:** 10.3390/life14121675

**Published:** 2024-12-18

**Authors:** Nathali Carvajal-Tello, Alejandro Segura-Ordoñez, Valeria Grisales-Jaramillo, Laura Isabella Rayo-Salazar, Katheryne Julieh Hernandez-Peñuela, Jose Luis Estela-Zape

**Affiliations:** 1 Health and Movement Research Group, Universidad Santiago de Cali, Cali 760001, Colombia; nathali.carvajal00@usc.edu.co (N.C.-T.); alejandro.segura00@usc.edu.co (A.S.-O.); valeria.grisales00@usc.edu.co (V.G.-J.); laura.rayo00@usc.edu.co (L.I.R.-S.); katheryne.hernandez00@usc.edu.co (K.J.H.-P.); 2 Physiotherapy Academic Program, Universidad Santiago de Cali, Cali 760001, Colombia; 3 Department of Anesthesiology and Resuscitation, Universidad del Valle, Cali 760015, Colombia; 4 Hospital Universitario del Valle, Cali 760032, Colombia; 5 Department of Biomedical Sciences, Universidad del Valle, Cali 760015, Colombia; 6 Department of Biomedical Sciences, Universidad Santiago de Cali, Cali 760001, Colombia

**Keywords:** bronchiolitis, syncytial virus, bronchial unobstruction, physiotherapy treatment, techniques

## Abstract

Background: Bronchiolitis is a seasonal viral infection of the respiratory tract that causes numerous childhood hospitalizations annually. Treatments vary based on severity, with mild cases requiring fluids and moderate to severe cases involving hospitalization with oxygen therapy, bronchodilators, and chest physiotherapy. Manual bronchial clearance techniques differ between Anglo-Saxon and European schools, and their effectiveness remains a subject of debate. Objective: The aim of this systematic review is to evaluate the effectiveness of manual bronchial clearance techniques in bronchiolitis by assessing clinical outcomes, including improved ventilation, increased oxygen saturation, and enhanced hemodynamic and respiratory stability. Materials and Methods: A systematic review was conducted between 2013 and 2024 using PRISMA guidelines. Databases searched included PubMed, Science Direct, Scopus, Springer, and Google Scholar; the inclusion criteria focused on randomized clinical trials and cohort studies in English, Spanish, and Portuguese. The selection bias was evaluated. The study was registered in Prospero (CRD42023486450). Results: Five articles involving 291 participants diagnosed with mild to moderate bronchiolitis were analyzed. The assessed techniques included Anglo-Saxon school and European School. Heart rate was evaluated in four studies, showing significant reductions in one (*p* < 0.01), while the significance in the other studies was not specified. Respiratory rate was assessed in three studies, with significant results being seen in two (*p* < 0.05). SpO2 was examined in all six studies, demonstrating significant improvements in two (*p* = 0.02 and *p* < 0.05). The Kristjansson respiratory score showed significant changes in one study (*p* = 0.005), and the Wang respiratory score indicated significant results in another (*p* = 0.03). These findings support the efficacy of chest physiotherapy techniques in managing bronchiolitis. Conclusions: While Anglo-Saxon techniques are widely used, their effectiveness remains a subject of debate. In contrast, European techniques indicate promising clinical outcomes, including improved ventilation, increased oxygen saturation, and enhanced respiratory stability; however, additional studies could further validate these findings.

## 1. Introduction

Acute bronchiolitis is a seasonal disease with a higher prevalence between autumn and spring. During the first year of life, its incidence ranges from 11% to 15% [1,2]. Annually, it causes approximately 199,000 deaths in children under two years of age and about one million hospitalizations, particularly in developing countries [3]. In developed countries, fatalities are rare and are primarily associated with comorbidities such as chronic lung or heart disease, neuromuscular disorders, and premature birth [4]. Respiratory syncytial virus (RSV) accounts for approximately 80% of bronchiolitis cases in infants under six months. Other common infectious agents include influenza viruses types I–III, rhinoviruses, adenoviruses, and metapneumovirus, which also contribute to the disease. These pathogens increase the risk for infants due to their smaller airways and immature immune systems. Identified risk factors include daycare attendance, lack of breastfeeding, parental smoking, low birth weights, and being aged under 12 months [5].

Inflammation and excessive bronchial mucus secretion are clinical manifestations resulting from a dysregulated inflammatory response in the bronchial epithelium caused by viral agents that can lead to airway obstruction. This obstruction reduces the diameter of the bronchi and bronchioles, impairing ventilation and gas exchange. Understanding these clinical manifestations is crucial for determining the appropriate management strategies for bronchiolitis [1].

The treatment of bronchiolitis is based on its severity. Mild cases are typically managed with fluid therapy to prevent dehydration, as respiratory congestion can interfere with feeding. Moderate to severe cases require hospitalization for continuous monitoring and respiratory support, which may include low-flow oxygen therapy or high-flow nasal cannula depending on the level of respiratory distress and oxygen saturation. Bronchodilators may be used to relieve bronchial smooth muscle obstruction, while corticosteroids help reduce bronchial inflammation. Respiratory physiotherapy supports secretion clearance, enhancing ventilation and alveolar–capillary gas exchanges [6].

Manual physiotherapy techniques differ by approach. The Anglo-Saxon school uses methods such as vibration, percussion, and Postural Drainage, while the European School employs techniques like Prolonged Slow Expiration (ELPr), Rhinopharyngeal Retrograde Clearance (RRC), Expiratory Flow Augmentation (EFA), autogenous drainage (AD), assisted autogenous drainage (AAD), and instrumental clearance techniques [7,8].

Anglo-Saxon techniques can cause complications, including irritability and gastroesophageal reflux, and their effectiveness in isolation is questionable, although in combination they have shown benefits in pathologies such as bronchiectasis [9]. On the other hand, European techniques, focused on flow mobilization, appear to provide relief in moderate cases of bronchiolitis, although more scientific evidence is needed to support efficacy [10,11].

Respiratory physiotherapy in bronchiolitis should be based on the severity of the disease and an individualized patient assessment. However, there are questions regarding its clinical application in mucociliary clearance and airway patency. These questions are particularly relevant when considering the differences between the practices recommended by the Anglo-Saxon and European Schools. Such differences reflect variations in the available evidence and treatment guidelines, which can impact the selection of interventions and their clinical effectiveness [12,13].

Therefore, the aim of this systematic review is to evaluate the effectiveness of manual bronchial clearance techniques in bronchiolitis by assessing clinical outcomes, including improved ventilation, increased oxygen saturation, and enhanced hemodynamic and respiratory stability.

## 2. Materials and Methods

The present systematic review was conducted in accordance with the guidelines outlined in the statement PRISMA (Preferred Reporting Items for Systematic Reviews and Meta-Analyses) [14]. Registration in prospero: CRD42023486450.

Sources of information search: Research was examined in relation to the study variables, and the following study types were included: randomized clinical trials (RCTs) and cohort studies (CSs) from 2013 to 2024. We also utlized reviews that met the needs of the search from the following electronic databases: PubMed, ScienceDirect, Scopus, Springer, and Google Scholar until 10 August 2024 in English, Spanish, and Portuguese.

The following MeSH terms were used for the search, and the different combinations were used with the following Boolean operators: (bronchiolitis OR bronchiolitis, viral) AND (clearance, mucociliary OR Mucociliary Transport OR Transport, Mucociliary) AND (Physical Therapy Modalities OR drainage, Postural OR Physical Therapy Techniques).

Review question: The following research question was formulated using the PICO strategy: what is the effectiveness of manual bronchial clearance techniques in the treatment of bronchiolitis? Additionally, the following elements were determined:

Participants/Population: Pediatric patients diagnosed with bronchiolitis.

Intervention/Exposure: Manual bronchial clearance techniques.

Control Group: Other respiratory techniques of bronchial clearance from the Anglo-Saxon or European School; instrumental techniques.

Results: Evaluation of pulmonary mechanics, oxygenation, and hemodynamic stability.

### 2.1. Eligibility Criteria

Inclusion criteria: Articles analyzed in pediatric patients with any clinical phenotype of bronchiolitis, where bronchial clearance techniques for the treatment of bronchiolitis were used, with any of the following techniques: vibration, percussion, High-Frequency Chest Wall Oscillation (HCFWO), Directed Cough (DC), Forced Expiratory Techniques (FETs), Active Cycle of Breathing Techniques (ACBTs), Postural Drainage (PD), Prolonged Slow Expiration (PSE), Rhinopharyngeal Retrograde Clearance (RRC), Provoked Cough (PC), and Assisted Autogenic Drainage (AAD). Articles and/or scientific evidence published between 2013 and 2024. Studies available in English, Spanish, and Portuguese. Only full-text articles.

Exclusion criteria: Studies that performed other bronchial clearance techniques such as instrumental techniques. Systematic review studies, case series, and case reports were not included.

Selection of records: An initial search using MeSH terms was carried out in the databases mentioned (see Appendix A), followed by selection of articles by filtering by year using an Excel matrix. Both the title and abstract were evaluated to determine the relevance of the articles to the research topic and whether they addressed the problematic question of the review. Duplicates found were eliminated. To collect specific information from the articles, such as type of study, presence of pediatric patients with bronchiolitis, use of manual bronchial clearance techniques, and effect measurements, another Excel matrix was used. In addition, the inclusion of each article and the presence of duplicates were recorded (see Appendix B).

Data extraction: Information from the selected articles was extracted by three investigators (VG, LR, KH) while other investigators (AS, NC, JE) corroborated the data. Agreements and disagreements on the content of the articles were discussed with the aim of identifying the effectiveness of bronchial clearance techniques in the treatment of bronchiolitis. Subsequently, the articles were reviewed in their entirety and relevant characteristics were analyzed for the extraction of relevant information.

Evaluation of the risk of bias (quality): After reviewing the information and discussing the agreements and disagreements on the selected articles, the quality of the articles was evaluated. In cases of discrepancy, the fifth and sixth authors (AS) (JE) were called to avoid selection bias. The PEDro [15] scale was used for RCTs and the New Castle Ottawa Scale (NOS) [16] for CSs. The PEDro [15] scale consists of 11 items, scoring 1 if met and 0 if not. Methodological quality was set as low, intermediate, or high according to the sum total. The NOS [16] uses a score to judge a study based on three perspectives: the selection of the study groups; the comparability of the groups; and the attainment of the exposure or outcome of interest for case–control or cohort studies. According to this scale, studies were classified into high- or low-quality studies using a score of 6 as the cut-off point.

Data synthesis: A detailed analysis of the study designs was conducted that examined the demographic characteristics of the population, sample size, data sources, and the languages used. The impact of bronchial clearance techniques in the treatment of bronchiolitis was also evaluated. To synthesize the data, we followed the SWIM guideline (Campbell et al., 2020) [17], which provides a clear structure for reports in intervention reviews. In addition, RevMan 5.4.1 software (The Cochrane Collaboration, 2020) [18] was used to perform meta-analyses in cases of homogeneous data, ensuring accurate presentation of the results with a 95% confidence interval (CI) [19].

### 2.2. Types of Interventions

#### 2.2.1. Anglo-Saxon School

- Vibration and percussion techniques: These involve the application of rapid compression or percussion to the thorax by the physiotherapist, generating an oscillation that helps to mobilize respiratory secretions [20].
- Postural Drainage (PD): Consists of adopting positions that follow the anatomy of the bronchial tree, thus facilitating the flow of secretions from the smallest to the largest branches, favoring their expulsion by means of gravity [20].

#### 2.2.2. European School

- Forced Expiratory Techniques (FETs) involve the manual application of external force on the rib cage to drain bronchial secretions and facilitate their expulsion, thus reducing their viscosity [21].
- Prolonged Slow Expiration (PSE) is a passive technique that applies slow manual thoraco-abdominal pressure from the end of the spontaneous expiration to the residual volume, indicated for infants and children up to 3 years of age, facilitating the displacement of bronchial secretions [22].
- Autogenous Drainage (AD) involves the patient’s control of breathing to move secretions from the middle and/or distal airways to the proximal airways, facilitating their elimination through expiratory airflows [22].
- Autogenic Assisted Drainage (AAD) is similar to AD but is used when the patient cannot perform AD independently, allowing them to be assisted by the physiotherapist with manual and verbal commands [22].
- Increased Expiratory Flow (IEF) involves a cephalocaudal compression performed on the lung bases of the thorax to mobilize secretions from distal to proximal airways, facilitating expectoration [23].

### 2.3. Main Results

#### 2.3.1. Pulmonary Mechanics

- A respiratory distress assessment instrument (RDAI) assesses the severity of the disease by analyzing clinical variables, with scores from 1 to 17, according to the location and intensity of retractions and wheezing [24].
- The Kristjansson respiratory score (KRS) evaluates five items, including respiratory rate, chest retractions, respiratory noise, skin color, and general condition. Each clinical sign is scored from zero to two, with a total score of 0 to 10, indicating greater severity increases [24].
- The Wang respiratory score (WRD) considers respiratory distress by assessing retractions, respiratory rate, saturation, and general condition. It is composed of four items, each scored from zero to three, except general status, which is scored as zero for normal or three for irritability or lethargy. The total score ranges from 0 to 12 [25,26,27].

#### 2.3.2. Oxygenation

- Oxygen saturation (SaO_2_) indicates the ratio of oxygen present in hemoglobin to the total amount of hemoglobin available [28].
- Arterial oxygen pressure (PaO_2_) reflects the rate of oxygen passing through the membrane of the alveolus and capillary, along with the elimination of carbon dioxide (CO_2_) [29].

#### 2.3.3. Hemodynamic Stability

- Heart rate (HR) is the number of times per minute that the heart contracts and relaxes, generating the pulsatile wave of blood [30].
- Respiratory frequency (RF) is the number of times a person breathes per minute [31].
- Blood pressure (BP) is the force exerted by the blood on the arterial walls [32].

## 3. Results

**Figure 1:**
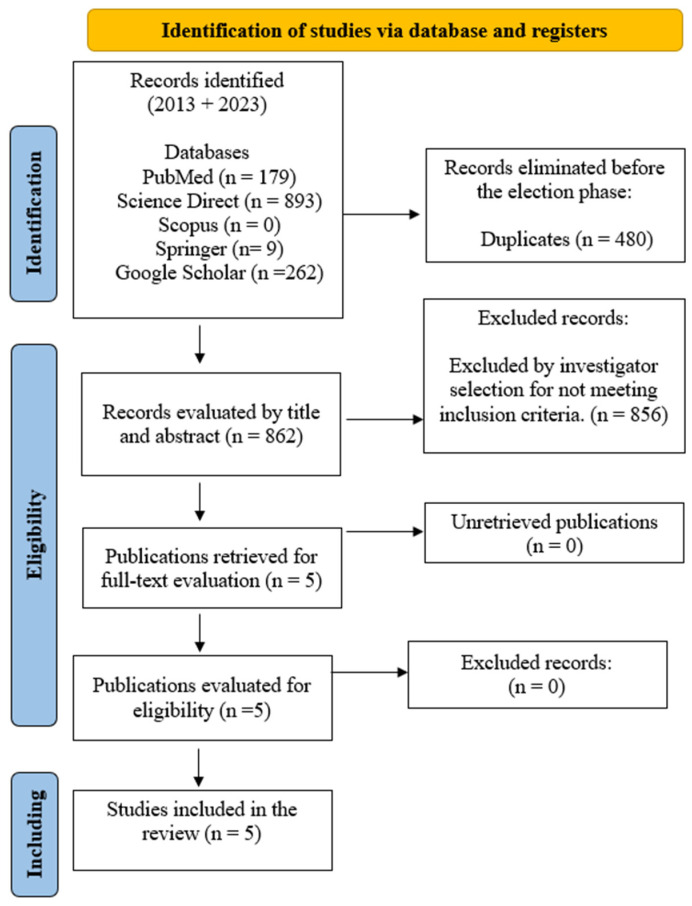
PRISMA flowchart.

The literature review allowed for the registration of 1343 studies from the five databases mentioned above; 480 records were eliminated before the duplicate choice phase, and 862 records were limited to 862 that were evaluated by title and abstract. Then, 856 were excluded for not meeting the inclusion criteria, leaving five studies for full-text reading. A total of five articles were selected for analysis in this systematic review (Figure 1).

### 3.1. Characteristics of the Bibliography

Of the five included studies, 60% n = (3) were found in the PubMed database, followed by 20% in Google Scholar n = (1), and 20% in Springer n = (1). Among them, four CCT 80% n = (4) and one CS 20% n = (1). Scimago Q1 quartile 40% n = (2), Q3 with 40% n = (2), Q4 with 20% n = (1). From the American continent 60% n = (3), Asia with 20% n = (1) and Europe with 20% n = (1). In the English language, 80% n = (4), and in Portuguese 20% n = (1). From the year 2014 (40%) n = (2), 2013 with 20% n = (1), 2017 with 20% n = (1), and 2021 with 20% n = (1) (Table 1).

### 3.2. General Characteristics of the Patients

The total number of participants was 291, aged between 29 days and 2 years, and the inclusion characteristics included a clinical diagnosis of mild or moderate bronchiolitis and a signature of consent from the patient responsible, while the exclusion characteristics included children with respiratory diseases associated with other diseases, diagnosis of severe bronchiolitis, and patients using any mechanical ventilation device (Table 2).

### 3.3. Description of the Interventions

The sessions per day ranged between one and five daily sessions, with the durations per session being between 10 and 20 min. The most used technique was Postural Drainage (PD) n = 3, followed by Provoked Cough n = 2, vibration, and percussion with equal proportion n = 2 (Table 3).

### 3.4. Evaluation of the Measurement Effect of Manual Bronchial Clearance Techniques

Table 4 shows the results of the measurements performed in relation to the variables HR, RR, SpO2, respiratory distress assessment instrument (RADAI), Kristjansson respiratory score (KRS), and Wang respiratory score (WRS) to evaluate the effect of chest physiotherapy techniques on bronchiolitis.

The main results are summarized below. HR was evaluated in three studies [35,36,37] without statistical significance. RR was assessed in two studies [35,36] where the *p* value was significant in one article [36]. The SpO2 was examined in all the studies included in this review, with one article reporting statistically significant results (*p* = <0.05) [35,36] after the intervention. In another two studies, the results were not significant (*p* = 0.082 [34] and *p* = 0.91 [37]), and in the remaining study [33], the *p*-values were not specified.

The RADAI was evaluated in a study [33] which does not specify its statistical significance. The KRS was evaluated in one study [34] where significant changes were found after the intervention *p* = 0.005. The WRS was evaluated in one study [37], in which it also had a significance value of *p* = 0.03 (Table 4).

### 3.5. Methodological Quality of the Studies

Four articles of this review described in Table 5 were evaluated with the PEDro15 scale, which determines the methodological quality of RCTs, obtaining a result of 75% n = (3) with high quality.

Of the five articles included in this review, one article described was evaluated with the NOS [16]; the study by Gonçalves et al., 2014 [36] included in our review was a non-comparative study that obtained a score of seven stars, reflecting a high methodological quality.

## 4. Discussion

The aim of the present systematic review was to identify the effectiveness of manual bronchial clearance techniques in the treatment of bronchiolitis. According to the results of five studies with a total of 291 participants, there is evidence that the techniques improve the child’s HR, RF, SpO2, muscle retractions, breath sounds, and skin color, using indices such as RDAI, KRS, and WRS. However, significant differences in the results were not found in all the studies reviewed, which suggests the need to deepen in this field with more representative samples to strengthen the scientific evidence supporting intervention with these techniques in bronchiolitis.

Bronchiolitis, characterized by inflammation, airway narrowing, and mucus production, presents with symptoms such as fever, dyspnea, cough, and shortness of breath, which can significantly impact a child’s quality of life [33]. Common symptoms include fever, dyspnea, cough, nasal obstruction, and shortness of breath. These symptoms can be severe and significantly affect the child’s quality of life [38]. Some studies have shown that chest physiotherapy techniques can improve parameters such as HR, FR, SpO2, RADAI index, KRS and WRS, helping to mobilize and expel secretions from airways [36].

In cases of bronchospasm, early administration of bronchodilators has been shown to relax bronchial smooth muscles, improve respiratory mechanics, and optimize gas exchanges, reducing the risk of complications [33]. However, in severe cases, studies [6,39] recommend prioritizing high-flow nasal cannula oxygen therapy in combination with bronchodilators. The use of manual bronchial clearance techniques further supports this approach by improving ventilation, enhancing oxygenation, and stabilizing respiratory function. Together, these pharmacological and non-pharmacological interventions provide an integrated strategy to optimize clinical outcomes, particularly in severe bronchiolitis.

Several studies, such as Rochat et al. (2012) [38], did not demonstrate benefits in regard to length of hospital stay, need for oxygen, or clinical improvement with the application of passive expiratory techniques, vibration, and percussion in severe bronchiolitis, although they did find significant changes when the intervention was complemented with oxygen therapy and pharmacology. Similarly, Sanchez M et al. (2012) [40] found that chest physiotherapy was not effective in reducing hospital stays or oxygen therapy time in patients with acute bronchiolitis; however, they did find that there was a reduction in oxygen hours in children with respiratory syncytial virus using nasopharyngeal ventilation.

Our review indicated that the Anglo-Saxon school’s chest physiotherapy techniques were the most widely implemented, specifically the DP technique [32,33,35], accounting for 66.6% of the studies published between 2013 and 2016. The effectiveness of these interventions has shown mixed results. Remondini et al. (2014) [33] found no statistically significant changes with the use of PD in children with bronchiolitis. More recent studies, such as that of Pinto et al. (2021) [34], focus on the European school, including techniques such as PSE, RC and PC, finding statistical significance in the evaluated variables SpO2 and the KRS respiratory score. Van Ginderdeuren et al. (2017) [37], who implemented AAD and HFCWO, found significant changes in the Wang respiratory score but not in HR and SpO2.

The prescription of instrumental techniques applied in children with bronchiolitis varies according to the severity of the disease and the type of patient. Rupavathy et al. (2016) [32] used HCFWO for 20 min, twice a day, while Ginderdeuren et al. (2017) [37] used HCFWO once a week with a frequency of 300 cycles/min and a pressure of between 6 and 10 mbar, observing efficacy in decreasing respiratory symptoms and hospital stay. Leemans et al. (2020) [41], in patients with cystic fibrosis (CF), noted that HCFWO is generally prescribed and effective at improving mucus transport, suggesting its potential usefulness in bronchiolitis and other respiratory pathologies.

In the review by Roqué et al. (2016) [20], immediate improvements in respiratory score were evidenced in patients with moderate bronchiolitis who received PSE. Pinto et al. (2020) [24] also found a positive and significant impact on the respiratory status of children with mild to moderate bronchiolitis using this technique. However, Jacinto et al. (2013) [35] did not present significant results when applying the technique of PC once a week. Ginderdeuren et al. (2017) [37] investigated the AAD technique, finding a significant reduction in hospital stays and respiratory symptoms when applying it once a day for 20 min.

On the other hand, techniques from the European School, such as PSE, AAD, and PSE, have demonstrated greater efficacy and tolerance in managing bronchiolitis, particularly in pediatric patients, compared to Anglo-Saxon techniques [20,42,43]. PSE, which involves controlled and extended expiration, aids in the mobilization of secretions, thereby reducing the severity of respiratory symptoms with less invasiveness and improved clinical acceptance [44,45,46].

PSE and RC further enhance these benefits by improving lung function and alleviating obstructions in both proximal and distal airways, with significant improvements being observed in clinical parameters such as HR, RR, and SpO2 [44,47]. In contrast, Anglo-Saxon techniques, such as vibration and manual percussion, face limitations due to their operational frequencies (4–8 Hz), which are suboptimal for providing the necessary stimulus to the airways, where a frequency exceeding 25 Hz is required for effectiveness [48]. Additionally, these techniques are often less tolerated in pediatric patients, which diminishes their effectiveness in bronchiolitis management [33,35,36,48].

When bronchiolitis presents with upper respiratory symptoms, such as hyaline rhinorrhea, RRC enhances upper airway clearance, which reduces the increase in anatomical dead space and minimizes respiratory effort, particularly in patients who breathe primarily through the nose [49,50]. Several studies suggest that combining RRC, nasopharyngeal secretion aspiration, and European School techniques improves SpO2, RR, HR, and clinical findings (*p* = 0.044) [38]. However, no significant reduction in hospital stay duration was observed in RSV-positive patients, although a progressive reduction in the need for supplemental oxygen was noted (*p* = 0.042) [40].

The controversy surrounding the efficacy of manual bronchial clearance techniques should not detract from the validity of the findings in the reviewed studies. Although some trials did not show significant improvements across all clinical variables, improvements in SpO2, RR, and symptom reduction suggest a positive impact. Variations in study design, methodology, and patient severity may explain the observed discrepancies in results. The integration of European School techniques, such as PSE, IEF, AAD, and PT, suggests that their effectiveness is likely influenced by the consistency of their application and their combination with other therapeutic approaches.

When selecting European School techniques for managing bronchiolitis, particular attention should be given to patients with bronchial hyper-responsiveness and excessive secretion production. Pulmonary auscultation plays a critical role in identifying lung sounds, such as rhonchi, which signal the presence of secretions in the bronchial tree. This can help differentiate between distal and peripheral secretions. Techniques that utilize low flows and volumes are effective in mobilizing secretions from the distal airways, while those with higher flows and volumes are more suited to mobilizing peripheral secretions. The combined use of both approaches optimizes mucociliary clearance, aiding in secretion removal and reducing airway obstructions.

Preliminary evidence indicates that manual bronchial clearance techniques, particularly those from the European School, may provide clinical benefits in bronchiolitis management. However, the existing studies are limited by small sample sizes and methodological variability, which reduce statistical power and generalizability. Differences in study design, patient populations, and treatment protocols further complicate direct comparisons. These limitations underscore the need for larger, more consistent studies with standardized methodologies to more definitively assess the effectiveness of these techniques and provide stronger evidence for clinical practice.

Future research should focus on larger studies to improve statistical power and generalizability. Standardizing methodologies will help minimize variability and enhance comparability across trials. Research should prioritize evaluating the individual and combined effects of European techniques such as PSE, IEF, AAD, and PT, especially in specific subgroups, such as patients with bronchial hyper-responsiveness or excessive secretion production. Comparative studies between European and Anglo-Saxon approaches are needed to establish their relative advantages, while further research into clinical outcomes—such as symptom reduction, oxygen saturation, and respiratory rate—will strengthen the evidence base for manual bronchial clearance in bronchiolitis management.

## 5. Conclusions

The European School of Bronchial Obstruction Manual Techniques demonstrates greater effectiveness in treating pediatric bronchiolitis compared to the Anglo-Saxon techniques. These methods improve secretion mobilization, lung function, and SpO2. However, the overall efficacy of chest physiotherapy in bronchiolitis remains uncertain due to inconsistencies in the literature.

To enhance clinical practice, it is recommended that European manual techniques such as PSE, RC and AAD are incorporated in managing bronchiolitis. Further studies with larger sample sizes and standardized protocols are necessary to validate these findings and establish optimal clinical practices.

## Figures and Tables

**Table 1:** Characteristics of the bibliography.

#	Authors	Title	Database	Journal	Scimago Quartile	Type of Study	Country	Continent	Language	Year	Objective
1	Remondini et al. [33]	Comparative analysis of the effects of two chest physiotherapy interventions in patients with bronchiolitis during the hospitalization period.	PubMed	Einstein	Q3	CCT	Brazil	America	Portuguese	2014	To evaluate and compare the effects of two interventions in patients hospitalized for acute bronchiolitis.
2	Pinto et al. [34]	Outpatient chest physiotherapy in acute bronchiolitis in children under two years of age.	Google Scholar	Hong Kong Physiotherapy Journal	Q3	CCT	China	Asia	English	2021	To analyze the role of a modern chest physiotherapy intervention versus no intervention on the respiratory status of children under two years of age with mild to moderate bronchiolitis.
3	Jacinto et al. [35]	Physiotherapy for airway clearance improves cardiac autonomic modulation in children with acute bronchiolitis. autonomic modulation in children with acute bronchiolitis.	PubMed	Brazilian Journal of Physical Therapy	Q1	CCT	Brazil	America	English	2013	To investigate the effects of conventional physical therapy for airway clearance and nasotracheal suctioning on heart rate variability in pediatric patients with acute bronchiolitis.
4	Gonçalves et al. [36]	Evaluation of physiological parameters before and after respiratory physiotherapy in newborns with acute viral bronchiolitis.	Springer	International Archives of Medicine	Q4	CS	Brazil	America	English	2014	To evaluate physiological parameters before and after respiratory therapy procedure in newborns with acute viral bronchiolitis.
5	Van Ginderdeuren et al. [37]	Efficacy of airway clearance techniques in hospitalized children with acute bronchiolitis.	PubMed	Pediatric Pulmonology	Q1	CCT	Belgium	Europe	English	2017	To evaluate the efficacy of two airway clearance techniques in children < 24 months hospitalized with mild to moderate bronchiolitis.

Abbreviations: Not specified (N/S), controlled clinical trial (CCT), cohort study (CS).

**Table 2:** General characteristics of the patients.

#	First Author, Year	n	EG/CG	Age (Years/Months)	Inclusion Characteristics	Exclusion Characteristics
1	Remondini et al. [33]	29	EG = 48 CG = 35	3–12 months	Clinical diagnosis of acute bronchiolitis, infants 3 to 12 months old	Uncorrected congenital heart disease, neuropathy, underlying ventilatory support lung disease, no parental or guardian agreement to participate in research.
2	Pinto et al. [34]	105	EG = 42 CG = 38	0–2 years	Children up to 2 years of age admitted to the Pediatric Emergency Department and diagnosed with acute bronchiolitis.	Severe bronchiolitis: 70 bpm or 50 bpm (in infants younger than six months or older, respectively), global retractions, apnea, nasal flaring, oxygen saturation (SpO2) of 88%, lethargy, dehydration and abnormal peripheral perfusion; need for hospital admission; and presence of comorbidities, namely prematurity, chronic pulmonary or neuromuscular disease, congenital heart disease, trisomy 21 or other congenital malformations.
3	Jacinto et al. [35]	24	EG = 12 CG = 12	2–11 months (with a mean age of 6 ± 2 months).	Children born at term	Age older than 12 months, presence of chronic cardiovascular or respiratory disease, prescription of vasoactive or sedative drugs, invasive mechanical ventilation, and children in contact isolation.
4	Gonçalves et al. [36]	30	EG = 30	29 days–6 months.	Sign the written consent of the patient’s responsible, with prescription of physiotherapeutic care, clinical and radiological diagnosis of bronchiolitis with confirmation and being in the acute phase of the disease.	Patients using any positive pressure ventilation device through a tracheal tube, nasal prong or face mask, under medication of vasoactive drugs, birth defects, cardiac disease, neurological disorders or genetic syndromes.
5	Van Ginderdeuren et al. [37]	103	CG = 36 EG1 = 33 EG2 = 34	Under 24 months	Eligible within 24 h of admission if they had mild to moderate bronchiolitis with a wang score of seventy.	Wang score < 3 and 18, comorbidities such as cystic fibrosis, neuromuscular or congenital cardiopathy, respiratory distress, requiring immediate ICU admission, gestational age < 34 weeks.

Abbreviations: Experimental Group (EG), Control Group (CG), Beats Per Minute (bpm), oxygen saturation (SpO2), Intensive Care Unit (ICU), Manual Hyperinflations (MHIs), Expiratory Flow Augmentation Technique (EFIT), and Percutaneous Intrapulhmonary Ventilation (IPV).

**Table 3:** Description of interventions.

#	First Author, Year	Type of Technique	Intensity	Sessions per Day	Number of Weeks	Session Duration in Minutes
1	Remondini et al., 2014 [33]	PD	It was performed in supine, lateral (left and right) and/or seated position for 10 min.	1–4 s	N/S	10
		Percussion	Tapping for 10 min in each position.			
2	Pinto et al., 2021 [34]	PSE	2–3 respiratory cycles	3–5 s	2	20
		RRC	Consecutive respiratory cycles			
		PC	Gently press the trachea at the level of the suprasternal notch at the end of inspiration.			
3	Jacinto et al., 2013 [35]	Percussion	N/S	1 s	N/S	N/S
		Vibration				
		PC				
		PD				
4	Gonçalves et al., 2014 [36]	Re expansion	N/S	1 s	N/S	14–20
		PD				
		Vibration				
5	Van Ginderdeuren et al., 2017 [37]	AAD	Gently and progressively, using the patient’s breathing pattern and stabilizing the abdominal wall.	1 s	N/S	20
		HCFWO	Frequency of 300 cycles/min and a pressure between 6 and 10 mbar.			

Abbreviations: Non-Specific (N/S), Experimental Group (EG), Control Group (CG), Positive Pressure Positive Expiratory Pressure Oscillatory Device (HCFWO), Directed Cough (DC), Forced Expiratory Techniques (FETs), Active Cycle of Breathing Technique (ACBT), Postural Drainage (PD), Prolonged Slow Expiration (PSE), Rhinopharyngeal Retrograde Clearance (RRC), Provoked Cough (PC), and Assisted Autogenic Drainage (AAD).

**Table 4:** Evaluation of the measurement effect of manual bronchial clearance techniques.

#	First Author, Year	Measurements	Basal				Posterior			
1	Remondini et al., 2014 [33]	SpO2	%	G1 (n)	G2	P	%	G1 (n)	G2 (n)	P
			<92	6 (12.5)	7 (20)	N/S	<92	5 (10.4)	3 (8.6)	N/S
			≥92%	42 (87.5	28 (80)		≥92%	43 (89.6)	32 (91.4)	
		RADAI		5.02 (2.07)	5.8 (1.51)	N/S		3.13 (1.81)	3.26 (1.96)	N/S
2	Pinto et al., 2021 [34]	KRS		EG	CG	P		EG	CG	P
			n	3.4 (1.3)	3.3 (1.3)	0.805	n	0.3 (0.5)	1.2 (1.5)	0.005
		SpO2	%	96 (2.085)	95.5 (1.505)	0.254	%	97.7 (1.517)	96.7 (2.144)	0.082
3	Jacinto et al., 2013 [35]	HR		EG	CG	P		EG	CG	P
			BPM	153 ± 14	143 ± 9	0.05	BPM	149 ± 10	142 ± 8	N/S
		RR	BPM	44 ± 6	42 ± 5	N/S	BPM	39 ± 7	38 ± 6	N/S
		SpO2	%	95 ± 3 *	98 ± 2	0.05	%	96 ± 2	97 ± 2	N/S
4	Gonçalves et al., 2014 [36]			EG		P		CG		P
		HR	BPM	142.4 ± 14		N/S	BPM	139.7 ± 14		N/S
		RR	BPM	62.1 ± 14			BPM	60.7 ± 12		<0.05
		SpO2	%	94.9 ± 1.5			%	97.3 ± 0.5		<0.05
5	Van Ginderdeuren et al., 2017 [37]		CG	EG(AAD)	GE(IPV)	P	CG	EG(AAD)	EG(IPV)	P
		HR	5 ± 10	3 ± 10	4 ± 13	0.68	6 ± 10	8 ± 8	7 ± 10	0.65
		SpO2	0 ± 1	−1 ± 1	−1 ± 1	0.07	−1 ± 3	0 ± 1	0 ± 1	0.91
		WANG	0.2 ± 0.3	0.5 ± 0.5	0.7 ± 0.5	0.04	0.5 ± 0.4	0.8 ± 0.6	0.9 ± 0.5	0.03

Abbreviations: Oxygen saturation (SpO2), Control Group (CG), Group 1 (G1), Group 2 (G2), Number of participants (n), heart rate (HR), respiratory rate (RR), respiratory distress assessment instrument (RDAI), Beats Per Minute (BPM), Breaths Per Minute (BPM), not specified (N/S), Kristjansson respiratory score (KRS), assisted autogenous drainage (AAD), Intrapulmonary Percussive Ventilation (IPV).

**Table 5:** Methodological quality of the controlled clinical trials studies (PEDro score).

First Author	1 *	2	3	4	5	6	7	8	9	10	11	Total	Methodological Quality
Remondini et al. [33]	─	1	1	1	0	0	0	1	1	1	1	7	Intermediate
Pinto et al. [34]	─	1	0	1	0	0	0	1	1	1	1	6	Intermediate
Jacinto et al. [35]	─	1	1	1	0	0	0	1	1	1	1	7	Intermediate
Van Ginderdeuren et al. [37]	─	1	1	1	1	0	0	1	1	0	1	7	High

PEDro scale criteria: (1) Choice criteria were specified (*—This item is not used to calculate the PEDro score). (2) Subjects were randomly assigned to groups (in a crossover study, subjects were randomly distributed as they received treatments). (3) Assignment was concealed. (4) Groups were similar at baseline with respect to the most important prognostic indicators. (5) All subjects were blinded. (6) All therapists administering therapy were blinded. (7) All assessors measuring at least one key outcome were blinded. (8) Measures of at least one of the key outcomes were obtained from more than 85% of subjects initially assigned to groups. (9) Results were presented for all subjects who received treatment or were assigned to the Control Group; when this was not possible, the data for at least one key outcome were analyzed by “intention-to-treat”. (10) Results of statistical comparisons between groups were reported for at least one key outcome. (11) The study provides point and variability measures for at least one key outcome. Abbreviations 1 = item met, 0 = item not met. Quality criteria: ≥7 high quality; 5–6 intermediate quality; and ≤4 low quality.

## Data Availability

No new data were created or analyzed in this study.

## Appendix A

**Table A1:** Data extraction and collection.

**Database: Google Scholar**				
**#**	**Search Method**	**Search Home**	**End of Search**	**Found Articles**
1	Bronchiolitis OR Bronchiolitis, Viral AND clearance, mucociliary OR Mucociliary Transport OR Transport, Mucociliary AND Physical Therapy Modalities OR Drainage, Postural OR Physical Therapy Techniques	4 October 2023	9 July 2024	262
**Database: PubMed**				
**#**	**Search Method**	**Search Home**	**End of Search**	**Found Articles**
1	Bronchiolitis AND clearance, mucociliary AND Physical Therapy Modalities OR Drainage, Postural	18 October 2023	10 August 2024	179
**Database: Springer**				
**#**	**Search Method**	**Search Home**	**End of Search**	**Found Articles**
1	Bronchiolitis AND clearance, mucociliary AND Physical Therapy Modalities OR Drainage, Postural	25 October 2023	25 July 2024	9
**Database: Science Direct**				
**#**	**Search Method**	**Search Home**	**End of Search**	**Found Articles**
1	Bronchiolitis AND clearance, mucociliary AND Physical Therapy Modalities OR Drainage, Postural	25 October 2023	25 July 2024	1120
**Database: Scopus**				
**#**	**Search Method**	**Search Home**	**End of Search**	**Found Articles**
1	Bronchiolitis AND clearance, mucociliary AND Physical Therapy Modalities OR Drainage, Postural	1 November 2023	1 July 2024	0

## Appendix B

**Table A2:** Data Selection Process.

No.	Reviewer	Title	Abstract	Type of Study	Bronchiolitis	Techniques	Effect	Included	Duplicate
									
